# Prevalence and associated health and lifestyle factors of myopic maculopathy in northern China: the Kailuan eye study

**DOI:** 10.1186/s12886-023-02896-3

**Published:** 2023-04-24

**Authors:** Haiwei Wang, Jinqiong Zhou, Ya Xing Wang, Qian Wang, Yanni Yan, Xuan Yang, Jingyan Yang, Wenjia Zhou, Zihan Nie, Xuhan Shi, Haixia Ji, Yongpeng Zhang, Xuehui Shi, Wen-Bin Wei

**Affiliations:** 1 Beijing Tongren Eye Center, Beijing key Laboratory of Intraocular Tumor Diagnosis and Treatment, Beijing Ophthalmology & Visual Sciences Key Lab, Medical Artificial Intelligence Research and Verification Key Laboratory of the Ministry of Industry and Information Technology, Beijing Tongren Hospital, Beijing Tongren Eye Center, Capital Medical University, Beijing, China; 2 Department of Ophthalmology, Fuxing Hospital, Capital Medical University, Beijing, China; 3 Beijing Institute of Ophthalmology, Beijing Key Laboratory of Ophthalmology and Visual Sciences, Beijing Tongren Hospital, Beijing Tongren Eye Center, Capital Medical University, Beijing, China

**Keywords:** Myopic maculopathy, Risk factors, Myopia

## Abstract

**Background:**

To evaluate the prevalence and associated health and lifestyle factors of myopic maculopathy (MM) in a northern Chinese industrial city.

**Methods:**

The cross-sectional Kailuan Eye Study included subjects who participated in the longitudinal Kailuan Study in 2016. Ophthalmologic and general examinations were performed on all the participants. MM was graded based on fundus photographs using the International Photographic Classification and Grading System. The prevalence of MM was evaluated. Univariate and multiple logistic regression were adopted to evaluated risk factors of MM.

**Results:**

The study included 8330 participants with gradable fundus photographs for MM and ocular biometry data. The prevalence of MM was 1.11% (93/8330; 95% confidence interval [CI] 0.89–1.33%). Diffuse chorioretinal atrophy, patchy chorioretinal atrophy, macular atrophy, and plus lesions were observed in 72 (0.9%), 15 (0.2%), 6 (0.007%), and 32 eyes (0.4%), respectively. MM was more common in eyes with longer axial length (OR 4.517; 95%CI 3.273 to 6.235) and in participants with hypertension (OR 3.460; 95%CI 1.152 to 10.391), and older age (OR 1.084; 95%CI 1.036 to 1.134).

**Conclusions:**

The MM was present in 1.11% of the northern Chinese individuals 21 years or older and the associate factors include longer axial length, older age, and hypertension.

## Backgroud

Myopia has been a public health problem worldwide, especially in Asian countries [1–4]. Several studies reported that myopia related blinding complications are the leading cause of irreversible visual impairment [5–7]. It was estimated that up to 55.7 million people will have visual impairment, and up to 18.5 million people will be blind, associated with myopic maculopathy (MM) worldwide in 2050 [8].

The reported prevalence of MM in adult populations ranges from 0.2–3.8%[9–16] in previous studies worldwide and was higher among people with high myopia ranging from 13.3–72.7% [10, 12–14]. The risk factors of MM reported in previous studies were older age [9, 10, 12, 13], greater myopic refractive error [9, 10, 12–15] and longer axial length (AL) [9, 13, 15]. However, other relevant demographic factors, such as gender, education level and lifestyle parameters, such as smoking status, alcohol consumption and physical activity have yet to be comprehensively investigated.

Thus, we aim to determine the prevalence and associated health and lifestyle factors of MM in the industrial city of northern China.

## Methods

### Participants and setting

The Kailuan Eye Study is a cross-sectional study included participants who had undergone ophthalmologic and general examinations from the longitudinal Kailuan Study. The study population included employees and retirees of a coal mining company (Kailuan Group Company). The community of Kailuan located in the city of Tangshan with approximately 7.2 million inhabitants. The Tangshan city is situated approximately 150 km southeast of Beijing and is a center of the coal mining industry. Using the data from the longitudinal Kailuan Study, cluster sampling was performed in this study. The details of the sampling frame and methods were described elsewhere [17]. At baseline, the study population consisted of 14,440 individuals with an age ranging between 21 and 97 years. The examination in the present study were performed repeatedly at 2-year intervals [17, 18]. The study followed the tenets of the Declaration of Helsinki. The present study was approved by the Medical Ethics Committee of the Beijing Tongren Hospital, and informed consent was obtained from the individuals after explanation of the nature and possible consequences of the study.

### Ophthalmological and body examinations

The ophthalmologic examinations [19] which described in detail previously included measurement of visual acuity, tonometry, slit-lamp assisted biomicroscope of the anterior segment of the eye, ocular biometry applying optical low-coherence reflectometry (Lenstar 900 Optical Biometer; Haag-Streit, Koeniz, Switzerland) for the determination of the central corneal thickness, anterior chamber depth, lens thickness and axial length (AL). Al the optical biometry measurement was performed three times repeatedly and the coefficient of variation for all biometric measurements were less than 0.1. Using a nonmydriatic fundus camera (CR6-45NM; Canon, Inc., Osta, Tokyo, Japan), Simultaneous stereoscopic 45° color fundus photographs centered on the optic disc and on the macula were taken for each eye. If the pupil diameter did not allow taking fundus photographs with sufficient photographic quality, we dilated the pupil medically by applying eye drops containing 0.5% tropicamide and 0.5% phenylephrine hydrochloride.

Body height and weight and the circumference of the waist and hip were measured. The body mass index (BMI) and waist–hip ratio was calculated. The smoking index was calculated by multiplying the number of cigarettes per day by the number of years of smoking.

Blood pressure was assessed with the participants sitting for at least 5 min. Blood samples were collected under fasting conditions to determine the blood glucose, high-density lipoprotein cholesterol (HDL-C), low-density lipoprotein cholesterol (LDL-C), triglyceride (TC), and total cholesterol (TG) concentrations [20].

For all participants, an interview was performed with standardized questions about known major systemic diseases and lifestyle parameters. Systemic diseases included hypertension, diabetes mellitus (DM), hyperlipidemia. Lifestyle parameters included smoking status, nature of job, and smart phone usage.

The diagnosis of DM was based on any of the following three criteria: measurement of the fasting blood glucose concentration of 7.0 mmol/L, a self-reported history of DM, or a history of medication with hypoglycemic agents.

The diagnostic criteria for hypertension were blood pressure of ≥ 140/90 mmHg, positive history of hypertension, or the use of antihypertensive drugs.

The diagnostic criteria for hyperlipidemia included hypercholesterolemia (TG ≥ 6.2 mmol /L), hypertriglyceridemia (TC ≥ 2.3 mmol/L), mixed hyperlipidemia (TG ≥ 6.2 mmol /L and TC ≥ 2.3 mmol/L), and low HDL-C levels (< 1.0 mmol/L). The definitions above have been described in detail in previous literature [20].

### Definitions and MM grading

High myopia was defined as AL equal to or more than 26.5 mm [12, 21–23] in this study. MM was graded among myopic participants using the International Photographic Classification and Grading System for MM [24]. In brief, MM was classified into five categories based on its severity: no myopic retinal degenerative lesion—category 0 (C0); tessellated fundus only—category 1 (C1); diffuse chorioretinal atrophy—category 2 (C2); patchy chorioretinal atrophy—category 3 (C3); macular atrophy—category 4 (C4). Additional lesions including lacquer cracks (LCs), Fuch’s spot and myopic choroidal neovascularisation, that is, ‘plus’ lesions, were also recorded. The presence of MM was defined as C2 or greater, and/or any additional lesions. To evaluate the interobserver agreement of two experienced ophthalmologists (Z.J.Q., W.H.W.), a test set with 100 images from 100 participants with high myopia (60 had C2 or greater MM) was used to test the ophthalmologists. The unweighted kappa was 0.81 for C0/C1, 0.77 for C2, 0.83 for C3, 0.85 for C4, 0.69 for LC, and 0.67 for Fuch’s spot. The two ophthalmologists interpreted each retinal photograph independently using the above-described criteria, and patients with unanimous diagnosis of MM were included in the study.

### Statistical analysis

The prevalence of MM with 95% confidence intervals (CIs) were evaluated. Pearson χ2 tests and Mann-Whitney U test were adopted to compare the characteristics between those participants with and without MM. Univariate and multiple logistic regression were adopted to evaluated risk factors for the presence of MM. Odds ratios (ORs) with 95% CIs were calculated. Visual impairment was classified into moderate (decimal: 0.1–0.3), severe (decimal: 0.05–0.1), blindness (decimal: 0.02–0.05) and severe blindness (decimal: less than 0.02). The influence of MM on visual acuity was also evaluated between participants with MM and without these disorders using Pearson χ2 tests. The statistical analysis was performed using SPSS software (version 21.0; IBM/SPSS, Chicago, IL, USA). A p value < 0.05 was regarded as statistically significant.

## Result

Only participants with gradable fundus photographs for MM and AL data were included in this study. In the baseline examination of 14,440 participants, 11,648 (80.7%) had gradable binocular fundus photographs. Among these participants, the data of AL was available in 8330 (71.5%) individuals, so that eventually 8330 participants were included in the study. There were 5477 men (50.1%) and 2853 women (49.9%) and the mean age was 51.6 ± 13.8(21 to 97 years) years.

**Fig. 1:**
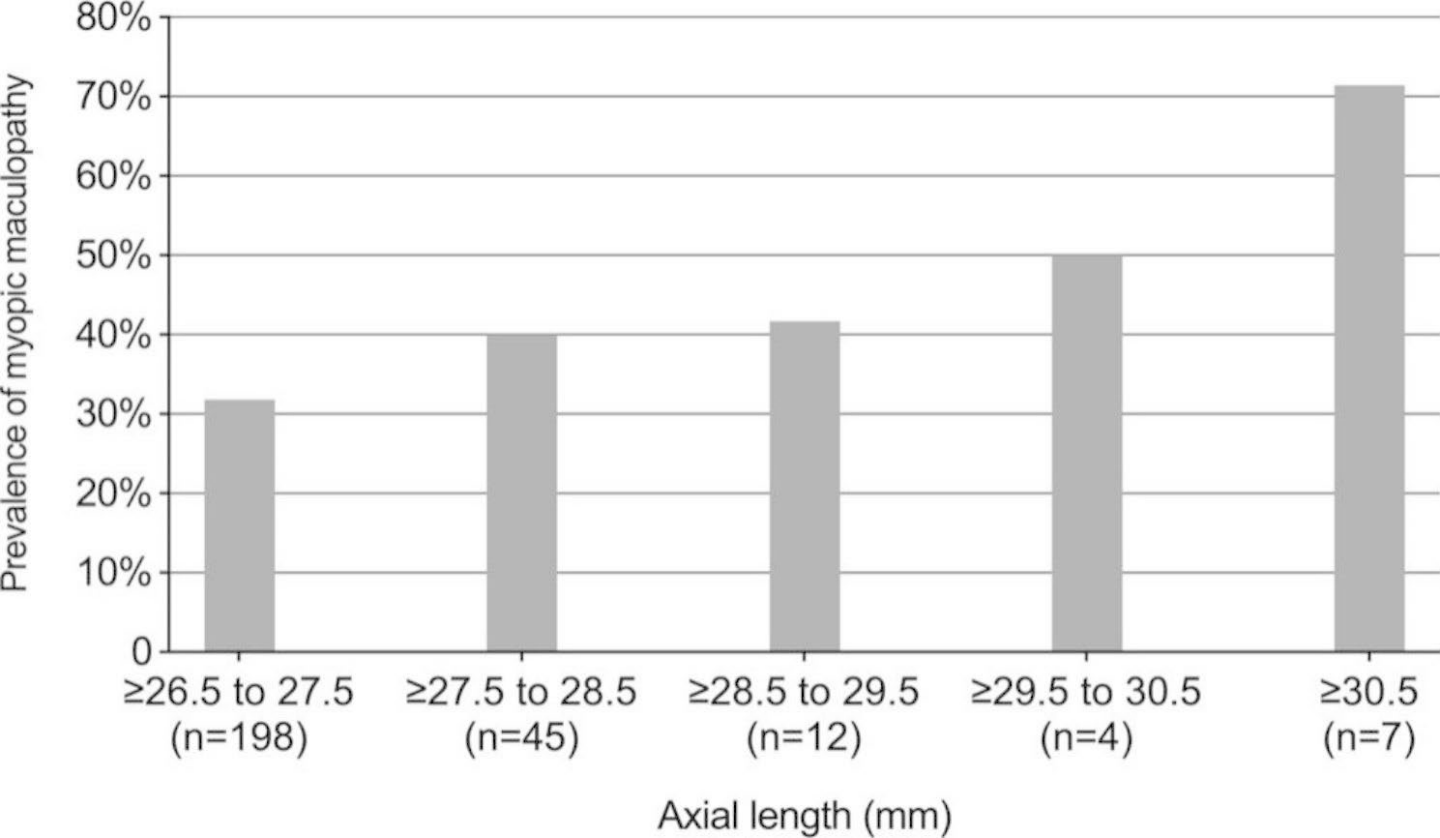
Prevalence of myopic maculopathy in relation to axial length (266 high myopic eyes)

**Fig. 2:**
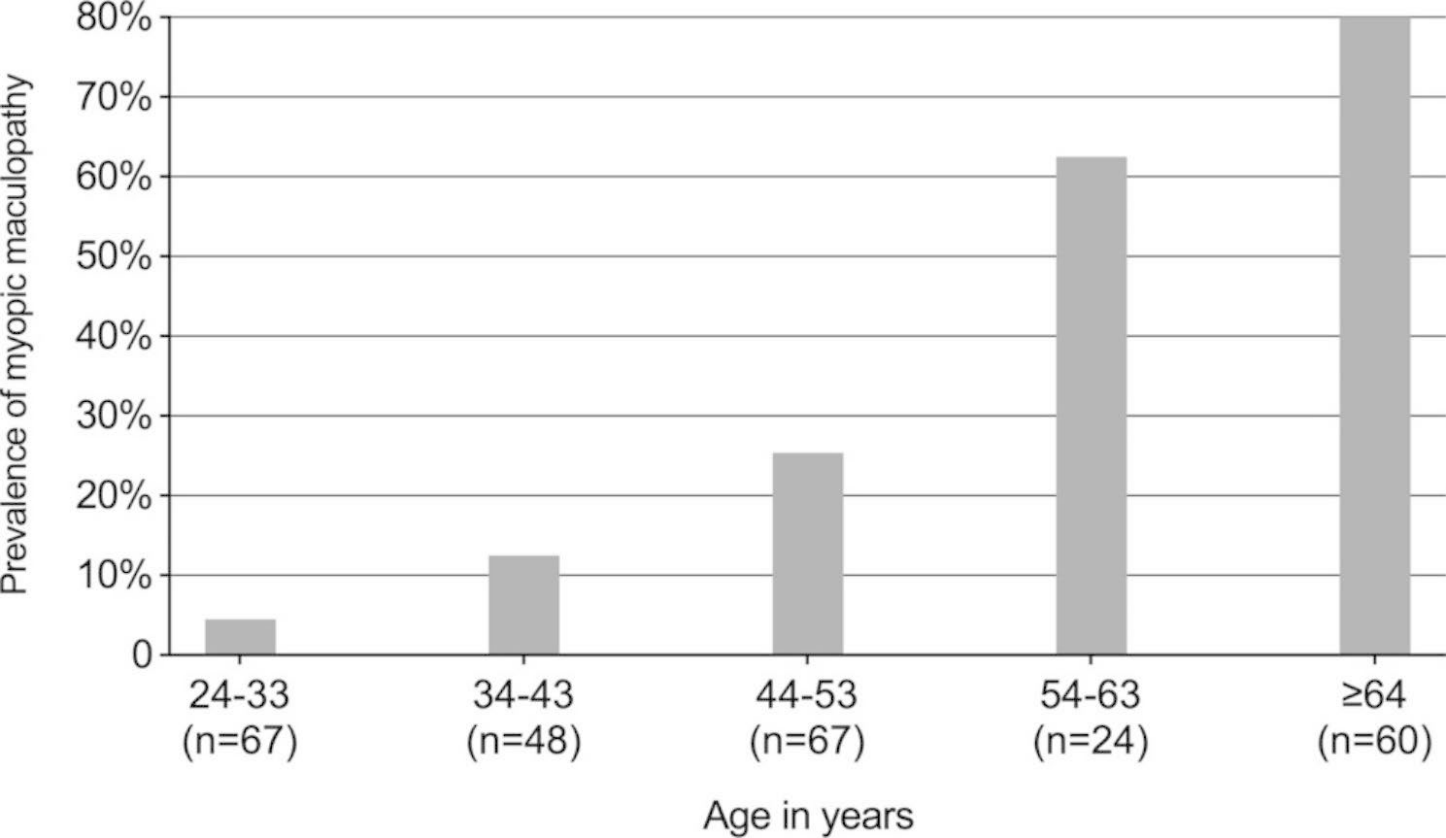
Prevalence of myopic maculopathy in relation to age (266 high myopic eyes)

The prevalence of high myopia was 3.19% (266/8330; 95% CI 2.81–3.57%) and the prevalence of MM was 1.11% (93/8330; 95% CI 0.89–1.33%) in the whole population. The prevalence of MM in high myopic participants was 34.96% (93/266; 95% CI 29.23–40.69%), and the MM prevalence increased with the AL (Fig. 1) and with the age (Fig. 2). A total of 125 MM lesions were identified in 93 eyes, including diffuse chorioretinal atrophy (n = 72), patchy chorioretinal atrophy (n = 15), macular atrophy (n = 6), and plus lesions (n = 32). The plus lesions included lacquer cracks (n = 20, 62.50%), Fuch’s spot (n = 7, 21.88%) and myopic choroidal neovascularization (n = 5, 15.62%).

Table 1 showed the age-specific and gender-specific prevalence of MM. The characteristics of eyes with or without MM (clinically significant MM) are shown in Table 2. Subjects with MM were older (62.4 ± 13.6 years vs. 51.6 ± 13.9, p < 0.001), had longer AL (27.41 ± 1.12 mm vs. 23.67 ± 1.15 mm, p < 0.001), had higher systolic blood pressure (144.5 ± 17.1 mmHg vs. 136.9 ± 22.6 mm, p = 0.001), had higher rate of hyperlipidemia (2.5% vs. 0.9%, p = 0.007), mental labor (1.5% vs. 0.7%, p = 0.009) and using smart phone (1.3% vs. 0.9%, p = 0.031) than those without MM. There was no statistically significant difference attributable to gender (p = 0.443), diastolic blood pressure (p = 0.383), body mass index (p = 0.863), waist-hip ratio (p = 0.894), smoking index (p = 0.960), and diabetes (p = 0.686) between participants with MM and those without MM.

**Table 1:** Age-specific and gender-specific prevalence of myopic maculopathy lesions

Group	At risk(n)	Category of myopic maculopathy					
		C0(%)	C1(%)	C2(%)	C3(%)	C4(%)	Plus lesions (%)
Age							
20–29	613	583 (95.1)	29 (4.7)	0 (0.0)	0 (0.0)	1 (0.2)	1 (0.2)
30–39	1258	1195 (95.0)	58 (4.6)	5 (0.4)	0 (0.0)	0 (0.0)	0 (0.0)
40–49	1611	1561 (96.9)	41 (2.5)	6 (0.4)	2 (0.1)	1 (0.1)	4 (0.2)
50–59	2106	2065 (98.1)	22 (1.0)	14 (0.7)	4 (0.2)	1 (0.0)	6 (0.3)
60–69	2007	1966 (99.5)	9 (0.4)	25 (1.2)	6 (0.3)	1 (0.0)	13 (0.6)
70–79	613	594 (96.9)	1 (0.2)	15 (2.4)	1 (0.2)	2 (0.3)	7 (1.1)
80+	122	111 (91.0)	0 (0.0)	7 (5.7)	2 (0.3)	0 (0.0)	2 (0.3)
Total	8330	8075 (96.9)	169 (2.0)	72 (0.9)	15 (0.2)	6 (0.0)	32 (0.4)
Gender							
female	2853	2775 (97.3)	50 (1.8)	17 (0.6)	6 (0.2)	5 (0.2)	15 (0.5)
male	5477	5300 (96.8)	119 (2.2)	55 (1.0)	9 (0.2)	1 (0.0)	17 (0.3)
Total	8330	8075 (96.9)	169 (2.0)	72 (0.9)	15 (0.2)	6 (0.0)	32 (0.4)

**Table 2:** Differences between the participants with myopic maculopathy and without myopic maculopathy

Parameters	Total	Myopic maculopathy		P value
		Present	Absent	
Age, years	8330	62.4 ± 13.6	51.6 ± 13.9	< 0.001
Gender(%)				
Male	5477	65(1.2)	5412(98.8)	0.443
Female	2853	28(1.0)	2825(99.0)	
AL, mm	8330	27.41 ± 1.12	23.67 ± 1.15	< 0.001
Systolic blood pressure, mmHg	5684	144.5 ± 17.1	136.9 ± 22.6	0.001
Diastolic blood pressure, mmHg	5684	81.4 ± 14.8	79.9 ± 10.6	0.383
Body mass index(BMI)	5691	24.9 ± 2.9	25.1 ± 3.5	0.863
Waist–hip ratio	5042	0.86 ± 0.12	0.87 ± 0.09	0.894
Smoking index	1407	252.8 ± 238.4	260.5 ± 429.2	0.960
Hypertension (%)				
No	5193	43(0.8)	5150(99.2)	0.006
Yes	677	13(1.9)	664(98.1)	
Diabetes (%)				
No	5480	51(0.9)	5429(99.1)	0.686
Yes	393	5(1.3)	388(98.7)	
Hyperlipidemia (%)				
No	5555	48(0.9)	5507(99.1)	0.007
Yes	314	8(2.5)	306(97.5)	
Nature of job (%)				
Physical labor	3351	22(0.7)	3329(99.3)	0.009
Mental labor	1058	16(1.5)	1042(98.5)	
Using of smart phone (%)				
No	1064	9(0.9)	1050(98.7)	0.031
Yes	2623	32(1.3)	2607(99.4)	

The univariate analysis in Table 3 involved the parameters which was significantly different in eyes with and without MM in Table 2. Table 3 showed the univariate logistic regression model which demonstrated that older age (OR 1.063; 95% CI 1.045 to 1.080), axial length (OR 5.473; 95% CI 4.419 to 6.777), systolic blood pressure (OR 1.007; 95% CI 1.001 to 1.012), hyperlipidemia (OR 2.999; 95% CI 1.407 to 6.396), mental labor (OR 2.324; 95% CI 1.216 to 4.441), and using smart phone (OR 0.460; 95% CI 0.224 to 0.946) were significantly associated with MM. Gender, body mass index, waist-hip ratio, smoking index, and diabetes did not correlate with the presence of MM.

**Table 3:** Univariate logistic regression analysis of risk factors associated with myopic maculopathy

Factors	Odds ratio	95% Confifidence Interval for OR	P value
Age, years	1.063	1.045, 1.080	< 0.001
Gender, men	1.212	0.776, 1.892	0.398
Axial length, mm	5.473	4.419, 6.777	< 0.001
Systolic blood pressure, mmHg	1.007	1.001, 1.012	0.017
Diastolic blood pressure, mmHg	1.013	0.989, 1.037	0.301
Body mass index (BMI)	0.983	0.911, 1.061	0.666
Waist–hip ratio	0.197	0.009, 4.314	0.303
Smoking index	1.000	0.998, 1.002	0.955
Hypertension	2.345	1.254, 4.383	0.008
Diabetes	1.372	0.544, 3.457	0.503
Hyperlipidemia	2.999	1.407, 6.396	0.004
Mental labor	2.324	1.216, 4.441	0.011
Using of smart phone	0.460	0.224, 0.946	0.035

The multivariable analysis in Table 4 included the presence of MM as dependent parameter and those variables as independent parameters that were associated with the presence of MM in the univariate analysis with a P value < 0.10.Table 4 showed the multiple logistic regression model which demonstrated that older age (OR 1.084; 95% CI 1.036 to 1.134), axial length (OR 4.517; 95% CI 3.273 to 6.235) and Hypertension (OR 3.460; 95% CI 1.152 to 10.391) were significantly associated with MM.

**Table 4:** Multiple logistic regression analysis of risk factors associated with myopic maculopathy

Factors	Odds ratio	95% Confifidence Interval for OR	P value
Age, years	1.084	1.036, 1.136	< 0.001
Axial length, mm	4.517	3.273, 6.235	< 0.001
Hypertension	3.460	1.152, 10.391	0.027
Hyperlipidemia	2.352	0.598, 9.252	0.221
Mental labor	1.963	0.666, 5.788	0.212
Using of smart phone	0.488	0.146, 1.624	0.242

Table 5 explores the impact of MM on visual impairment and blindness. Among 93 participants with MM, only 17 participants (18.3%) showed normal vision for presenting visual acuity; the rest (n = 76, 81.7%) had visual impairment or blindness, including 43 participants (46.2%) who had moderate visual impairment, 20 (21.5%) had severe visual impairment and 13 (14.0%) had blindness.

**Table 5:** Influence of myopic maculopathy on visual impairment and blindness

Present visual acuity	Total	Myopic maculopathy		P value
		Present (%)	Absent (%)	
Normal vision (≥ 0.3)	7138	17 (18.3)	7123 (88.3)	< 0.001
Moderate visual impairment (0.1–0.3)	937	43 (46.2)	893 (11.1)	
Severe visual impairment (0.05–0.1)	21	20 (21.5)	37 (0.5)	
Blindness (0.02–0.05)	7	8(8.6)	12 (0.1)	
Severe blindness (< 0.02)	13	5 (5.4)	3 (0.0)	
Total	8161	93 (100)	8068 (100)	

## Discussion

The overall prevalence of MM (1.11%) in this industrial city population was comparable with previous literature, such as that of the Yangxi Eye Study in south China (1.2%, N = 4469, aged ≥ 50 years), Handan Eye Study in north China(0.9%, N = 6603; aged ≥ 30 years), Hisayama Eye Study in Japan (1.7%, N = 1892; aged ≥ 40 years), and Blue Mountains Eye Study in Australia (1.2%, N = 3583; aged ≥ 49 years) [11, 13, 14, 25], but lower than that reported from the the Beijing Eye Study in north China(3.1%, N = 4319; aged ≥ 40 years) and Shihpai Eye Study in Taiwan (3.0%, N = 1058, aged ≥ 65 years) [10, 12]. Different age compositions in these study samples may influence the prevalence findings. In addition, the definition of MM among different studies were not consistent. These two important factors may contribute to the relative lower prevalence of current study [16]. Table 6 shows the comparison of prevalence of MM in different studies.

The risk of MM is higher in persons of older age, regardless of myopia severity and AL, which places MM as an age-related degenerative disease [26–28]. This is consistent with our findings, reporting an increase of prevalence with higher age (OR = 1.084 per year). Although MM occurs more often in older age, younger age may also be affected, which reported by Koh et al. in 593 high myopic eyes with a mean age of 21 years [29]. The greater prevalence of MM at an early age might reflect the genetic-driven early manifestations and early progression to high myopia in Asian young man may pose an additional risk factor for MM. Congruently, Chen et al. reported that the pattern of myopic lesions was age-specific: younger age was associated with lacquer cracks (OR 0.96, 95% CI 0.95 to 0.98, p < 0.001), while diffuse and patchy chorioretinal atrophy, macular atrophy and choroidal neovascularisation were more prevalent in older age [30].

In our study, the definition of AL was a prerequisite for myopic maculopathy cases. This approach increased the specificity for myopia-related maculopathy and diminished the issue in subjects with ambiguous fundus findings, and thus reduced ascertainment bias. Increasing AL was associated with a higher prevalence of myopic maculopathy in our study, with an OR of 4.517 comparing eyes with AL 26.5 mm or more compared with less than 26.5 mm. Our results mirrored other studies reporting an increase of MM with longer AL in a dose–response manner [9–11, 13, 15, 31]. Axial myopia is consistently held responsible for the development of myopic maculopathy [32].

The general health status and lifestyle habits parameters were also considered in the current study and the results showed that the risk of MM associated with hypertension. The Shihpai eye study reported an association with high blood pressure [12] which is consistent with our findings. Recent studies showed that systolic blood pressure played a significant role on the choroidal thickness change in normal subjects [33, 34]. Higher systolic blood pressure was associated with thinner choroidal thickness [34]. The high blood pressure in myopic patients may further compromise the already thinner choroid [35], interfere with the choroidal circulation, and hence increase the severity of maculopathy. Our finding of hypertension as risk factors needs further verification in longitudinal cohort studies. Future interventional studies or clinical studies might consider including blood pressure measurement for analysis. However, we could not find association of Diabetes, serum lipids, BMI, smoking, nature of job (mental or physical), and using smart phone (near distance working) with MM after multivariable adjustment in the present study. These findings are consistent with the results from Wong et al [36].

**Table 6:** Comparison of prevalence of myopic maculopathy in different studies

Studies	Year examined	Population	Participants	Age	Definition	Prevalence % (95%CI)	Risk factors
The present study	2016	Urban Chinese	8330	21–97	Diffuse chorioretinal atrophy	1.1(0.89–1.33)	Older age
					Patchy chorioretinal atrophy		Longer Axial length
					Macular atrophy		Hypertension
					Lacquer cracks		
					Fuch’s spot		
					Choroidal neovascularisation		
Yangxi Eye Study	2014	Rural Chinese	4469	≥ 50	Diffuse chorioretinal atrophy	1.4(1.0-1.8)	Older age
					Patchy chorioretinal atrophy		Higher refractive error
					Macular atrophy		
					Lacquer cracks		
					Fuch’s spot		
					Choroidal neovascularisation		
					Staphyloma		
Handan Eye Study	2006–2007	Rural Chinese	4409	≥ 50	Chorioretinal atrophy	1.2(0.9–1.6)	Older age
					Fuch’s spot		Higher refractive error
					Lacquer cracks		
					Staphyloma		
Beijing Eye Study	2001	Urban Chinese	4319	≥ 40	Chorioretinal atrophy	3.1(2.6–3.6)	Higher age
					Fuch’s spot		Worse visual acuity
					Lacquer cracks		Deeper anterior chamber
					Staphyloma		Larger optic disc
Shipai Eye Study	2000	Urban Chinese	1361	≥ 65	Deep chorioretinal atrophy	4.2(3.0-5.5)	High systolic blood
					Lacquer cracks		
					Geographic atrophy		
					Choroidal neovascularisation		
Gutenberg Health Study	2007–2012	Rural and urban Germany	519	35–74	Diffuse chorioretinal atrophy	10.3(7.9–13.3)	Older age
					Patchy chorioretinal atrophy		Higher refractive error
					Macular atrophy		Male gender
					Lacquer cracks		
					Fuch’s spot		
					Choroidal neovascularisation		
Hisayama Study	2005	Southern Japanese	1892	≥ 40	Diffuse chorioretinal atrophy	1.7(1.2–2.4)	Older age
					Patchy chorioretinal atrophy		Femal gender
					Macular atrophy		Longer Axial length
					Lacquer cracks		
Blue Mountains Eye Study	1992–1994	Australian	3654	≥ 49	Chorioretinal atrophy	1.2(0.9–1.6)	
					Lacquer cracks		
					Fuch’s spot		
					Staphyloma		

It has been demonstrated that MM was the leading cause of bilateral visual impairment and blindness [2–4, 28]. Our study mirrored this link between MM and visual impairment and blindness, with 81.7% (76/93) of participants with MM having a present visual acuity classified as visual impairment or blindness. Subjects with MM had significantly poorer visual acuity than subjects without MM(P < 0.001).

Strengths of this study include large sample size, the use of International Photographic Classification and Grading System for MM, reasonable response rates (71.5%), and standardized methodology for data collection, ocular biometry assessment, and fundus photography. Our study was limited in several aspects. On account of unable to acquire enough data of their spherical equivalent, we used a strict definition for high myopia (axial length ≧ 26.5 mm). This cutoff was adopted in several studies as the definition of high myopia [12, 21–23]. The prevalence of high myopia might have been underestimated to some extent. However, we believe the effect of including the axial length as a defining characteristic in the prevalence of MM is minimal if any. Furthermore, studies showed that there were significantly more retinal complications in eyes with axial length longer than 26.5 mm [21, 37–39]. As we did not perform OCT imaging, we cannot state on the ATN classification system [23] in which not only the atrophic (A) and neovascular (N) components of pathological myopia were included, but also the aspect of traction (T).

## Conclusions

In summary, the prevalence of clinical MM was present in 1.11% of the Chinese people at age 21–97 years in Kailuan area in northern China and 34.96% of high myopic people. MM was associated with longer axial length, with older age, and with hypertension.

## Data Availability

The datasets used and/or analysed during the current study available from the corresponding author on reasonable request.

## Abbreviations

MM: myopic maculopathy
AL: axial length
BMI: body mass index
HDL-C: high-density lipoprotein cholesterol
LDL-C: low-density lipoprotein cholesterol
TC: triglyceride
TG: total cholesterol
DM: diabetes mellitus
CI: confidence interval
OR: Odds ratio.
